# Mitofusin 2 Integrates Mitochondrial Network Remodelling, Mitophagy and Renewal of Respiratory Chain Proteins in Neurons after Oxygen and Glucose Deprivation

**DOI:** 10.1007/s12035-022-02981-6

**Published:** 2022-08-13

**Authors:** Piotr Wojtyniak, Anna Boratynska-Jasinska, Karolina Serwach, Joanna Gruszczynska-Biegala, Barbara Zablocka, Jacek Jaworski, Maria Kawalec

**Affiliations:** 1 Mossakowski Medical Research Institute, Polish Academy of Sciences, Warsaw, Poland; 2 International Institute of Molecular and Cell Biology, Warsaw, Poland

**Keywords:** Mitochondria, Mitophagy, Mitofusin 2, Primary neurons, Mitochondrial biogenesis, Mitochondrial DNA, Brain ischemia

## Abstract

In attempts to develop effective therapeutic strategies to limit post-ischemic injury, mitochondria emerge as a key element determining neuronal fate. Mitochondrial damage can be alleviated by various mechanisms including mitochondrial network remodelling, mitochondrial elimination and mitochondrial protein biogenesis. However, the mechanisms regulating relationships between these phenomena are poorly understood. We hypothesized that mitofusin 2 (Mfn2), a mitochondrial GTPase involved in mitochondrial fusion, mitochondria trafficking and mitochondria and endoplasmic reticulum (ER) tethering, may act as one of linking and regulatory factors in neurons following ischemic insult. To verify this assumption, we performed temporal oxygen and glucose deprivation (OGD/R) on rat cortical primary culture to determine whether Mfn2 protein reduction affected the onset of mitophagy, subsequent mitochondrial biogenesis and thus neuronal survival. We found that Mfn2 knockdown increased neuronal susceptibility to OGD/R, prevented mitochondrial network remodelling and resulted in prolonged mitophagosomes formation in response to the insult. Next, Mfn2 knockdown was observed to be accompanied by reduced Parkin protein levels and increased Parkin accumulation on mitochondria. As for wild-type neurons, OGD/R insult was followed by an elevated mtDNA content and an increase in respiratory chain proteins. Neither of these phenomena were observed for Mfn2 knockdown neurons. Collectively, our findings showed that Mfn2 in neurons affected their response to mild and transient OGD stress, balancing the extent of defective mitochondria elimination and positively influencing mitochondrial respiratory protein levels. Our study suggests that Mfn2 is one of essential elements for neuronal response to ischemic insult, necessary for neuronal survival.

**Supplementary Information:**

The online version contains supplementary material available at 10.1007/s12035-022-02981-6.

## Introduction

Mitochondria are a key determinant in proper neuronal functioning. Mitochondrial dynamics include mitochondrial fission, fusion and motility. The molecular mechanisms and critical mediators in mitochondrial dynamism have been discussed in minute detail and have been the subject of many detailed reviews [1–3]. The disturbances in the mitochondrial quality control mechanisms which affect the elimination of impaired mitochondria and consequently, mitochondrial turnover, are implicated in various neurodegenerative conditions, like Parkinson’s and Alzheimer’s Disease [4]. Mitochondria are also involved in neuronal response to the brain ischemia [5]. The precise mechanisms conditioning neuronal survival under ischemic stress are not entirely clear yet. To date, considerable effort has been put into better understanding of the role of mitochondria in neuronal response to the ischemic insult in order to find potential therapeutic targets to limit post-ischemic neuronal damage.

Impaired mitochondria become the source of pro-apoptotic stimuli, therefore, various endogenous mechanisms can occur to alleviate mitochondrial damage. An increased mitochondrial fusion and fission events have been shown to facilitate mixing of the mitochondria content thereby preventing the accumulation of mitochondrial defects [1], while severely damaged mitochondria were reported to be eliminated by mitophagy [6]. Finally, the activation of mitochondrial biogenesis was shown to restore mitochondrial content and bioenergetic abilities of the neurons [7, 8]. However, the mechanisms orchestrating mitochondrial dynamics, mitophagy and mitochondrial biogenesis are still poorly understood.

Among the variety of mitochondrial proteins, an outer mitochondrial membrane GTPase, mitofusin 2 (Mfn2), emerges as a unique player which integrates various functions, particularly mitochondrial fusion, mitochondrial movement, the tethering of mitochondria to the endoplasmic reticulum (ER) and, presumably, mitophagy.

Two mitofusins, Mfn1 and Mfn2, are known as mammalian homologues of yeast Fzo fusion protein [9]. Mfn1 and Mfn2 share high similarity in their molecular structures and, partly, their functions. By forming homo- and heterodimers, they juxtapose mitochondria and facilitate the outer mitochondrial membrane (OMM) fusion [10]. However, Mfn2 homodimers alone are weakly efficient to induce OMM fusion thereby making Mfn1 participation an obligatory factor [11]. By contrast, it is Mfn2, not Mfn1, that is present at the endoplasmic reticulum conditioning mitochondria-ER tethering [12] and ER morphology [13].

Mfn2 participates in mitophagy by interacting with key proteins mediating mitochondria elimination. The selective elimination of mitochondria via mitophagy was first described by Kim et al. (2007) [14]. It was further demonstrated that the disruption of mitochondrial membrane potential resulted in the accumulation of PTEN-induced kinase 1, PINK1, in OMM [15] and the subsequent recruitment of E3 ubiquitin ligase Parkin into the impaired mitochondria. Parkin, in turn, by ubiquitination of OMM proteins, facilitates the attachment of autophagosome membranes to the damaged mitochondria that undergo degradation [16]. As shown by Chan et al. (2013), Mfn2 phosphorylation by PINK1 facilitates the translocation of Parkin from cytosol to the impaired mitochondria causing PINK1-phosphorylated Mfn2 to serve as a receptor for Parkin [17]. Mfn2 undergoes Parkin-mediated ubiquitination [18] but the precise role of this phenomenon is still under investigation. Nonetheless, what has already been proven is that Parkin-mediated Mfn2 ubiquitination affects mitochondria and ER contact sites [19] while Parkin-mediated Mfn2 degradation in proteasome facilitates the dissociation of mitochondria from the ER thereby enabling the onset of mitophagy [20]. Consequently, the maintenance of mitochondria-ER contacts suppresses PINK1/Parkin-mediated mitophagy [21], which confirms the functional link between mitochondria-ER interaction and mitophagy.

At some point, the sites of mitochondria-ER contacts appear to be important for mitochondrial biogenesis as well. It was shown that Dynamin-related protein 1 (Drp1)-mediated mitochondrial division is crucial for appropriate mitochondrial nucleoid distribution during mitochondrial biogenesis [22] and that, at the same time, mitochondrial division preferentially occurs at mitochondria-ER contacts [23].

The role of other proteins mediating mitochondrial network remodelling after ischemic insult has been investigated before in in vitro and in vivo models. As shown by Wappler et al. (2013), the contribution of particular fusion and fission proteins, like Mfn1/2, Opa1, Drp1 and Fis1, varies depending on the duration of the insult, but the maintained fusion emerges as a predominant response of surviving neurons [24]. At the same time, in many experimental models the enhanced mitochondrial fission after ischemic insult was observed to precede apoptotic neuronal death [25], while the inhibition of Drp1-mediated mitochondrial fission turned out to be neuroprotective [26–28].

The precise molecular mechanism which regulates mitophagy and mitochondrial biogenesis has not yet been described, however, several links have already been observed. According to recent findings, the mutual antagonism between the PINK1/Parkin pathway and Peroxisome proliferator-activated receptor gamma coactivator 1-alpha (PGC-1α) expression can be detected [29]. PGC-1α is considered a master regulator for mitochondria biogenesis. PGC-1α works upstream of nuclear respiratory factor 1 (NRF-1), which is responsible for the expression of most respiratory complex proteins, and upstream of mitochondrial transcription factor A (TFAM), which is essential for mitochondrial DNA maintenance and replication [30]. It was further demonstrated that both Parkin and PINK1 deficiencies contribute to the accumulation of Parkin-interacting substrate (PARIS), which, in turn, inhibits PGC-1α expression [31, 32]. However, further research is needed to understand the complexity of this phenomenon.

In our study, we hypothesized that neuronal Mfn2 may act as a linking protein that integrates mitochondrial network remodelling with mitophagy and mitochondrial biogenesis. We further assumed that this interplay might be crucial for mitochondrial homeostasis and thus neuronal survival under ischemic insults. Our assumptions were based on the available scientific data on the role of Mfn2 in mitochondrial network dynamics (mitochondrial fusion and trafficking), the ability of Mfn2 to tether mitochondria and ER, and on the recorded fact that these Mfn2 functions can be modified by E3 ubiquitin ligases, such as Parkin, implicated in mitochondrial elimination.

To verify this concept, we performed a temporal oxygen and glucose deprivation (OGD), which is a well-established cellular model of ischemia-reperfusion injury [24, 33], on the primary culture of rat cortical neurons. We compared the effects of oxygen and glucose deprivation followed by reperfusion (OGD/R) on mitochondrial network morphology, mitophagosome formation as well as mitochondrial biogenesis markers in wild-type and Mfn2 knockdown neurons. In addition, we attempted to determine whether the knockdown of the Mfn2 may affect the onset of mitophagy and subsequent OGD/R-induced mitochondrial biogenesis, and eventually, the neuronal survival.

## Materials and Methods

### Primary Cell Cultures

Cortical neuronal cultures were prepared from Wistar rat brains on embryonic day 19 (E19). Pregnant female Wistar rats were provided by the Animal House of the Mossakowski Medical Research Institute, Polish Academy of Sciences (Warsaw, Poland). Animal care was provided in accordance with the European Communities Council Directive (86/609/EEC).

Embryo cortices were dissected and cut into 1-mm pieces on a 35-mm Petri dish in cold Ca^2+^/Mg^2+^-free HBSS (Gibco, Thermo Scientific, Grand Island, NY, USA). Then, the tissue was transferred to a 15-ml Falcon tube, rinsed twice with cold Ca^2+^/Mg^2+^-free HBSS and incubated for 10 min at 37 °C in Ca^2+^/Mg^2+^-free HBSS containing 0.2 % trypsin (Gibco). It was followed by double washing with cold HBSS in the presence of Ca^2+^/Mg^2+^ (Gibco). Next, the cell suspension was generated by passaging 10 times through a fire-polished glass Pasteur pipette in cold Ca^2+^/Mg^2+^ HBSS containing 1 mg/mL DNase I (Roche, Basel, Switzerland). The suspension was passed through a 70-μm cell strainer (Corning, USA) to eliminate tissue debris. For the immunoblotting, neurons were seeded on Poly-D-Lysine 6-well plates (Corning) at a density of 1 × 10^6^ cells/well. For fluorescence measurements, cells were seeded on Poly-D-Lysine coated 24-well plates (Corning) at a density of 2.2 × 10^5^ cells/well.

Neurons were seeded in a Neurobasal medium (Gibco) supplemented with 2% B-27 (Gibco), 0.5 mM GlutaMAX (Gibco), 12.5 μM glutamate (Merck/Sigma-Aldrich, Poznan, Poland), and 1% Antibiotic-Antimycotic (Gibco) at 37 °C in a humidified atmosphere with 5% CO_2_. On day in vitro (DIV) 2 half of the medium was replaced with glutamate-free growth medium containing non-neuronal cell proliferation inhibitor CultureOne Supplement (Gibco). Such neuronal culture was found to consist of more than 90% of neurons with a minimum number of non-neuronal cells, based on MAP2/GFAP immunofluorescence staining.

### Lentiviral Production and Cell Transduction

To induce Mfn2 knockdown, commercially available 29mer short-hairpin RNA (shRNA) constructs in lentiviral GFP vector (Origene, Rockville, USA, TL712567) were used. Two constructs, sh-Mfn2 B and sh-Mfn2 D, were selected for the experiments, based on satisfactory efficiency in Mfn2 reduction with a relatively low toxicity. As a negative control, a non-effective 29-mer scrambled shRNA cassette in pGFP-C-shLenti Vector was used (scrRNA; Origene). Viral production was performed as described in Gruszczynska-Biegala et al. (2020) [34]. Neurons were infected with sh-Mfn2 and scrRNA- carrying lentiviruses on DIV4 with a viral infection efficiency exceeding 90%. The experiments were performed 6 days post transduction (DIV 10).

### Oxygen–glucose Deprivation and Reoxygenation (OGD/R)

OGD/R was performed on DIV 10. For OGD/R treatment, OGD media, composed of 130 mM NaCl (Sigma-Aldrich, Saint Louis, USA), 2.5 mM KCl (Sigma-Aldrich), 2.2 mM CaCl_2_ (AppliChem GmbH, Darmstadt, Germany), 1.5 mM MgCl_2_ x 6H_2_O (AppliChem GmbH), 10 mM Hepes (AppliChem GmbH), pH 7.3–7.4, was bubbled with 95% N_2_/5% CO_2_ for 15 min.

The cells were washed twice with OGD media, then immediately transferred into the Modular Incubator Chamber (Billups-Rothenberg, San Diego, USA) filled with mixed gas containing 95% N_2_/5% CO_2_ for 15 min at 15–20 L/min. Thereafter, the sealed chamber was incubated at 37°C for 45 min reaching the total time of OGD as 1 h. Neurons in the control group were maintained under normoxic incubation conditions. After OGD, the cells were removed from the chamber, refreshed with previously collected conditioned culture medium and incubated at 37°C in 5% CO_2_ for 3 h or 24 h of the reperfusion phase.

### Immunoblotting

For western blotting, neurons were washed with PBS (Sigma-Aldrich) and lysed with Cell Lysis Buffer (Cell Signaling Technology, Danvers, USA) containing 1 mM PMSF (Sigma-Aldrich) for 5 min on ice. The samples were sonicated and centrifuged at 14,000 × g for 10 min at 4°C. The supernatant was collected, and a Modified Lowry Protein Assay (Thermo Scientific, Grand Island, NY, USA) was performed to determine the total protein concentration. The samples were diluted in a reducing sample buffer and boiled at 100°C for 5 min. For western blotting with anti-OXPHOS antibody cocktail (Abcam, Cambridge, UK), samples were not boiled, following the manufacturer’s guidelines.

Equal amounts of protein (20–35 μg) were analyzed by 10–12% SDS-PAGE, electro-transferred onto nitrocellulose (Amersham; Cytiva, USA) or Immobilon-P PVDF (Merck Millipore, Burlington, USA) membrane and stained for the total protein (Ponceau S staining). Following imaging of the total protein, the membranes were blocked with 5% non-fat milk in Tris-buffered saline (TBS) with 0.1% Tween 20 (TBST) for 1 h at room temperature. Thereafter, the membranes were incubated with the appropriate primary antibodies diluted in TBST or 2.5% milk/TBST at 4°C overnight, including anti-Mfn1 (1:1000, 11E9-1H12, Novus Biologicals, USA, NBP1-71775), anti-Mfn2 (1:1000, Sigma-Aldrich, M6319), anti-Opa1 (1:1000, D6U6N, Cell Signaling, 80471), anti-Drp1 (1:1000, 4E11B1, Cell Signaling, 14647), anti-TOM20 (1:500, Cell Signaling, Danvers, USA, 42406), anti-HSP60 (1:1000, Cell Signaling, 12165) anti-PRK-8 (1:250, Santa Cruz Biotechnology, Dallas, USA, sc-32282), anti-PGC-1α (1:250, Santa Cruz Biotechnology, sc-518025), anti-NRF1 (1:500; Proteintech, UK, 12482-1-AP), anti-NRF1 (1:250; Santa Cruz Biotechnology, sc-28379), anti-OXPHOS (1:500; Abcam, Cambridge, UK, ab110413). The membranes were washed three times for 5 min in TBST and incubated with the following peroxidase-conjugated secondary antibodies: anti-mouse (Sigma-Aldrich, A9044) or anti-rabbit (Sigma-Aldrich, A0545) diluted in 5% non-fat milk in TBST for 30 min at room temperature, and again washed for 5 min in the TBST. Bound antibodies were visualized by Amersham ECL or Amersham ECL Select detection reagent (Cytiva, USA). Blots were imaged and quantified using the Fusion FX imaging system (Vilber Lourmat, Marne-la-Vallée, France). The band intensities of the proteins of interest were normalized to the total protein densities corresponding to the same lane and quantified using ImageJ software with gel analyzer feature (NIH, Bethesda, MD, USA) as shown by Thacker et al. (2016) [35].

### Immunofluorescence and Image Acquisition

Neuronal cultures were seeded at a density of 7.9 × 10^4^/cm^2^ on 1.5 laminin coated coverslips (Neuvitro Corporation, Vancouver, WA, USA). The cells were stained with 100 nM Mitotracker Red CMXRos (Thermo Scientific) in preconditioned culture medium for 45 min., fixed with 4% paraformaldehyde (AppliChem, Darmstadt, Germany) in PBS (Sigma-Aldrich) followed by permeabilization with 0.25% Triton-X (Carl Roth, Karlsruhe, Germany) in PBS. The cells were stained with primary antibodies: mouse anti-Parkin (1:50; Santa Cruz Biotechnology, Santa Cruz, CA, sc-32282), mouse anti-MAP2 clone AP20 (1:250; EMD Millipore Corporation, Temecula, CA, USA, MAB3418) or rabbit anti-GFAP (1:1000; Abcam, Cambridge, UK, ab7260), detected by secondary antibodies: goat anti-mouse CF 633 (1:400; Sigma-Aldrich, SAB4600333), donkey anti-mouse Alexa Fluor 488 (1:400; Jackson ImmunoResearch, West Grove, PA, USA, cat. 715-545-150) or donkey anti-rabbit RR-X (1:400; Jackson ImmunoResearch, cat. 711-295-152). The nuclei were visualized with Hoechst 33342 (8 μM, Thermo Scientific). The samples were mounted in the ProLong Glass Antifade Mountant (Thermo Scientific).

The immunostained cultures were viewed under Zeiss LSM780 Axio Observer confocal microscope (Carl Zeiss AG, Oberkochen, Germany). Images were acquired using a 100× Alpha Plan-Apochromat oil immersion objective (1.46 NA). The unidirectional scanning mode was used and the image resolution was 1024 × 1024 pixels. When needed, at least 9 z-sections of 290–300 nm size per image were taken. Laser power and detector gain values were set once and repeated throughout the experiments.

### Quantitative Image Analysis of Mitochondrial Morphology and Parkin Accumulation

The acquired images were analyzed using the Mito-Morphology macro by Dagda et al. (2009) [36] created for ImageJ software (NIH, Bethesda, MD, USA). GFP fluorescence or MAP2 staining images were used to help draw outlines of the cell bodies, and these selections were copied into images with Mitotracker Red-stained mitochondria and processed by the Mito-Morphology macro. The method described by Van Laar et al. (2015) [37] was adapted to assess cells positive for Parkin accumulation on mitochondria. To confirm that Parkin colocalized with mitochondria in observed puncta, a line was drawn across the punctum and a plot of the fluorescence intensity along the line was examined, as shown previously [38]. Sites of Parkin accumulation displayed overlapping signals of high intensity both for Mitotracker and anti-Parkin channels. The neurons which exhibited at least one such punctum were considered positive, counted and related to the total number of imaged neurons in particular experimental time. The obtained data are the average of at least three independent experiments. The minimal number of cells analyzed for each experimental point was 20.

### Mitophagy Assay

Mitophagy Dye (Dojindo EU GmbH., Munich, Germany) [39] was used to visualize mitophagosomes in live cells according to the manufacturer’s instructions with minor modifications. In brief, the cells were plated on Ibidi μ-slide 8 Well plates (Ibidi GmbH, Germany) at a density of 8 × 10^4^ cells/well. On DIV 10, the neurons were incubated with 200 nM Mitophagy dye at 37 °C for 30 min, washed twice and subjected to OGD/R experiment. At the appropriate time before microscopic analysis, the cells were stained for 30 min with 1:1000 Lysoview 633 (Biotium, Inc., Fremont, CA, USA) and 8 μM Hoechst 33342 (Thermo Scientific) diluted in culture medium. The cells were washed twice and imaged using a Zeiss spinning disk Axio Observer Z1 confocal microscope (Carl Zeiss AG, Oberkochen, Germany) equipped with an incubation chamber with the 37°C temperature and humidified 5% CO_2_ atmosphere. The images were acquired as Z-stacks with 240 nm spacing using a 63× Plan Apo 1.4 objective. The image processing was performed with ImageJ software (NIH, Bethesda, MD, USA). The selection of Mitophagy dye- and LysoView-positive objects was made with Colocalization Highlighter plugin, which resulted in an 8-bit colocalized point images, further transformed into a binary images. The number of colocalized puncta was quantified by Analyze Particles feature, using the Size and Circularity parameters to exclude noise and occasional aggregates.

### DNA Isolation

Total cellular DNA was purified using E.Z.N.A. MicroElute Genomic DNA Kit (Omega Bio-tek, Norcross, GA, USA). In brief, at the indicated time points, the cells were washed twice, scraped from the plates, centrifuged at 1000 × g, suspended in PBS (Sigma-Aldrich) and processed according to the manufacturer’s protocol. The purity (absorbance ratio at 260/280) and concentration of DNA samples were determined spectroscopically using DeNovix DS-11 FX+ (DeNovix Inc., Wilmington, DE, USA).

### mtDNA Copy Number Quantitation

A singleplex real-time PCR assay was established for measuring the amount of rat mtDNA relative to the nuclear DNA. This assay targets the mitochondrial ND1 gene (26193) and the nuclear single-copy gene [40], β-actin (V01217.1) Primers and probes were designed using the Primer3web (https://primer3.ut.ee/) [41]. The probes were labeled at the 5′-end with FAM. A quencher dye, 6-carboxytetramethylrhodamine (TAMRA), was linked to the 3′-end of both probes. Primers and probes were synthesized by DNA Sequencing and Synthesis Facility IBB PAS, Warsaw, Poland. We used the following primers and probes: mtDNA forward primer, ND1226F: ACCCTCTCCCTTACACTAGC; mtDNA reverse primer, ND1405: AAGAGATGGTTTGGGCAACG; TaqMan probe, ND1_380TM: 5′-ACTCCCTATTCGGAGCCCTACGAGC; nuclear DNA forward primer, ACTB_F: GGGATGTTTGCTCCAACCAA; nuclear DNA reverse primer, ACT_R: GCGCTTTTGACTCAAGGATTTAA; TaqMan probe, ACTB_TM: 5′-CGGTCGCCTTCACCGTTCCAGTT. Real-time PCR was performed in a MicroAmp EnduraPlate optical 96-well reaction plate (Applied Biosystems, Foster City, CA, USA) sealed with MicroAmp optical adhesive film (Applied Biosystems) on the ABI 7500 FAST Real-time PCR System (Applied Biosystems). The reaction mix (total volume 20 μL) consisted of: 10 μL TaqMan Fast Advanced Master Mix (Applied Biosystems), 4 μL Nuclease-Free Water (Applied Biosystems), 2 μl mtDNA or nDNA primers (900 nM each), 2 μl mtDNA or nDNA probe (250 nM), 2 μl DNA (50 ng). The real-time PCR reactions were performed in triplicate for both genes. The temperature program was initiated with a polymerase activation at 95 °C for 2 min, followed by 40 cycles at 95 °C for 3 s and 60 °C for 30 s. The cycle threshold (Ct) values were determined using SDS 2.3 software (Applied Biosystems). Relative copy number was calculated using analysis of the difference in Ct between mtDNA and nuclear DNA. Relative quantification was performed by the ΔΔCt method [42] and expressing the ratio as a percentage of the calibrator — untreated control cells — set as 100%.

### RNA Extraction and RT-qPCR

Total RNA was extracted from cells using Total RNA Mini Plus Concentrator kit (A&A Biotechnology, Gdansk, Poland). For assessing RNA quality and yield, A260/A280 and A260/A230 ratios for RNA preparation samples were analyzed with a DeNovix DS-11 FX+ spectrophotometer (DeNovix Inc., Wilmington, DE, USA). To determine Parkin mRNA expression, reverse transcription was carried out using High-Capacity RNA-to-cDNA Kit (Applied Biosystems) with total RNA (2 μg) according to the manufacturer’s instructions. Real-time PCR was performed in triplicates using TaqMan Gene Expression Assay probes for Parkin (Rn00571787_m1), Gapdh (Rn01775763_g1) and β-actin (Rn01412977_g1) with TaqMan Fast Advanced Master Mix (Applied Biosystems) in the ABI 7500 FAST Real-time PCR System (Applied Biosystems). The thermal cycling was initiated by the polymerase activation step for 2 min at 95 °C, followed by 40 cycles at 95 °C for 3 s and 60 °C for 30 s. mRNA levels of β-actin or Gapdh were used as an internal control to normalize the mRNA levels of Parkin. The expression levels for Parkin were assessed in relation to Gapdh and β-actin expression (ΔΔCt method, according to Livak and Schmittgen (2001) [42]).

### Lactate Dehydrogenase (LDH) Assay

Neuronal death was examined by measuring LDH release into the culture medium, using CytoTox-ONE Homogenous Membrane Integrity Assay (Promega, Madison, WI, USA), according to the manufacturer’s manual. LDH levels for samples were normalized to Maximum LDH Release control.

### Pyknotic Nuclei Count

Control and OGD/R-subjected live neurons were incubated on DIV 10 and DIV 11 respectively, with 8 μM Hoechst 33342 (Thermo Scientific) in culture medium. After 30 min, the staining solution was replaced with HBSS (Gibco) and images were taken using Olympus IX71 fluorescence microscope equipped with Olympus Colorview III camera (Olympus, Tokyo, Japan) and dedicated Cell^F software. Nucleus counting was performed using the Cell Counter plugin for ImageJ (NIH, Bethesda, MD, USA).

### Mitochondrial Membrane Potential

Mitochondrial membrane potential was measured fluorometrically using JC-1 dye (5,5′,6,6-tetrachloro-1,1′,3,3-tetraethylbenzimidazolylcarbocyanine iodide; Sigma-Aldrich/Merck), according to Cossarizza et al. (2001) [43]. JC-1 fluorescence was measured at Ex/Em: 475/530 nm for green and at 475/590 nm for the red channel using monochromator-based microplate reader, Tecan INFINITE M1000 PRO with dedicated software (Tecan Group Ltd, Mannedorf, Switzerland). In brief, the neurons were cultured on 24-well BioCoat plates (Corning). On DIV 10, directly after the OGD experiment, control and OGD-treated neurons were incubated for 10 min with 1 uM JC-1. After washing, the fluorescence was measured in PhenolRed-free Ca^2+^/Mg^2+^ HBSS (Thermofisher Scientific, USA). In parallel, JC-1 measurement was performed on neurons treated for 1 h with 5–10 μM mitochondrial uncoupler CCCP (Carbonyl cyanide 3-chlorophenylhydrazone; Sigma-Aldrich) to further diminish the mitochondrial membrane potential.

### Statistical Analysis

The results are presented as mean ± standard deviation. Statistical analysis was performed using one-way analysis of variance (ANOVA) followed by the Bonferroni’s multiple comparison test. For data sets that did not meet the normal distribution, the Kruskal-Wallis test was used followed by Dunn’s multiple comparison test. All calculations were performed using GraphPad Prism 5.0 (GraphPad Software, San Diego, CA, USA).

## Results

### Mfn2 Knockdown Results in Increased Neuronal Damage 24 h after Oxygen and Glucose Deprivation

**Fig. 1:**
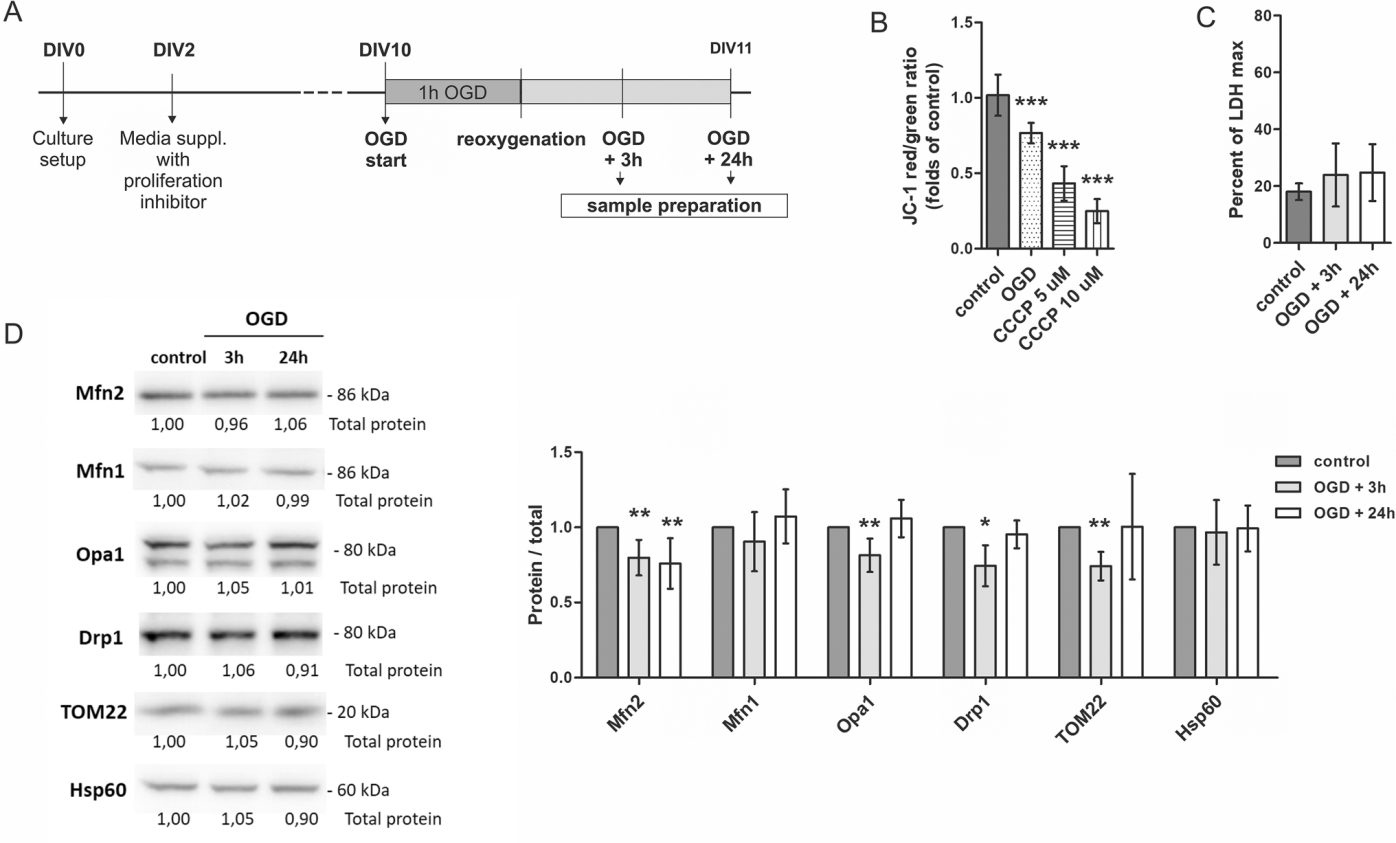
Mfn2 protein level is reduced after mild OGD in rat cortical neurons. (A) Experimental design. OGD — oxygen and glucose deprivation. DIV — day in vitro; (B) Effects of 1 h OGD on mitochondrial membrane potential in wild-type (wt) neurons expressed as relative red to green fluorescence ratio of JC-1 probe, directly after the insult. CCCP was used as a control of Δψ reduction. Mean ± SD; *n* = 4. ***p < 0.001 *vs* wt control; (C) Effects of OGD/R on cell survival in wt neurons measured as LDH release. Mean ± SD; *n* = 6. (D) Representative western blots and densitometric analysis of Mfn2, Mfn1, Opa1, Drp1, TOM22 and Hsp60 in cell lysates of wt neurons after OGD/R. *n* = 4. *p < 0.05, **p < 0.01, vs wt control. The optical density of the particular bands was normalized to the total protein in line stained with Ponceau S. Normalization factors are shown under representative western blot image (Supplementary Fig. 1)

The in vitro experiments were performed on primary cultures of rat cortical neurons with reduced numbers of glial cells. To mimic ischemic insult, neurons were subjected to the temporal deprivation of oxygen and glucose performed according to the scheme shown in Fig. 1A. After 10 days of culturing, the culture medium was replaced by deoxygenated glucose-free buffer and culture plates were placed in an oxygen-free chamber at 37°C. After 1 h, the culture medium was restored and neurons were returned to normoxic conditions (reoxygenation). The samples were collected at 3 h and 24 h. The controls were obtained from the neurons that were kept under normoxic condition throughout the experiment.

The duration of oxygen and glucose deprivation was optimised to avoid significant cell death 24 h later to allow for the observation of long-time effects of the insult. In our study, 1 h OGD reduced the mitochondrial membrane potential in wild-type (wt) neurons by almost 20 %, as shown by changes in JC-1 probe fluorescence (Fig. 1B). In comparison, 1-h treatment with the mitochondrial uncoupler, 5 μM CCCP, reduced the mitochondrial membrane potential in wt neurons by half, confirming the moderate character of the OGD insult (Fig. 1B). Also, for such a subtle insult, we did not observe an increase in LDH release in wt neurons (Fig. 1C).

As demonstrated by western blot, 1-h OGD significantly and constantly reduced the Mfn2 level in total cell lysates of wt neurons. The reduction of the Mfn2 protein was observed as early as 3 h after the insult, while the Mfn1 and Hsp60 proteins were unaltered and Opa1, Drp1 and TOM22 temporarily dropped at 3 h after the OGD (Fig. 1D and Supplementary Fig. 1).

**Fig. 2:**
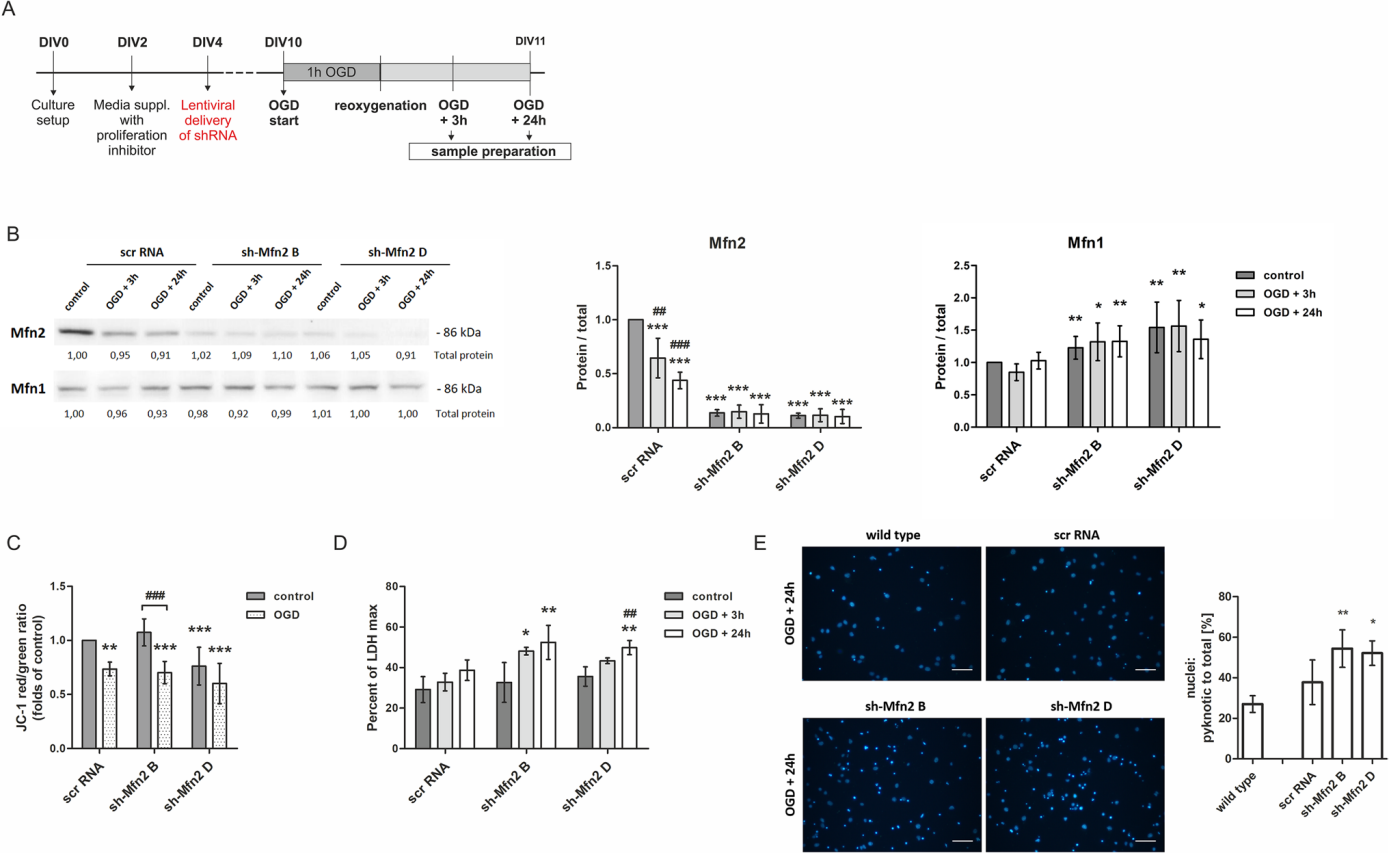
Mfn2 knockdown increases the susceptibility of neurons to OGD/R. (A) Experimental design. OGD — oxygen and glucose deprivation. DIV — day in vitro; (B) Representative western blot and densitometric analysis of Mfn1 and Mfn2 in cell lysates of shRNA-transduced neurons after OGD/R. *n* = 4. * *p* < 0.05, **p < 0.01, ***p < 0.001 vs scrRNA control; ##*p* < 0.01; ###*p* < 0.001 vs shRNA control; the optical density of the particular bands was normalized to the total protein in line stained with Ponceau S. Normalization factors are shown under representative western blot images. (C) Effects of 1 h OGD on mitochondrial membrane potential in shRNA-transduced neurons expressed as relative red to green fluorescence ratio of JC-1 probe, directly after the insult. Mean ± SD; *n* = 4. **p < 0.01, ***p < 0.001 vs scrRNA control; ###*p* < 0.001 vs sh-Mfn2 control; (D) Effects of OGD/R on cell survival measured as LDH release. Mean ± SD; *n* = 6. *p < 0.05; **p < 0.01 vs scrRNA control. ##*p* < 0.01 vs sh-Mfn2 control; (E) Effect of OGD/R on the percentage of pyknotic nuclei in the total number of nuclei in wt and shRNA-transduced neurons, 24 h after OGD. Nuclei were stained with Hoechst 33342; chart and representative images. Scale bars: 50 μm, *n* = 4. *p < 0.05, **p < 0.01 vs scrRNA control

To determine whether Mfn2 protein affects neuronal damage after OGD, first, we validated the knockdown constructs for Mfn2 (sh-Mfn2 B and sh-Mfn2 D), as demonstrated at Fig. 2A–B. As shown by western blot, both shRNA constructs significantly reduced the level of Mfn2, while the level of the Mfn1 did not diminish. As measured on DIV10, the sh-Mfn2 B and sh-Mfn2 D constructs reduced Mfn2 protein level by 86% and 89%, respectively, relative to scrRNA-transduced neurons. Meanwhile, Mfn1 protein was unaltered by scrRNA and OGD, while in sh-Mfn2 neurons Mfn1 was elevated by 23% (sh-Mfn2 B) or 54% (sh-Mfn2 D) before the insult, and up to 33% (sh-Mfn2 B) and 56% (sh-Mfn2 D) after the insult (Fig. 2B).

The reduction in the mitochondrial membrane potential caused by 1 h OGD in shRNA-transduced neurons was comparable to those observed for wt neurons (Fig. 2C).

To verify whether Mfn2 deficiency can affect neuronal viability, we measured lactate dehydrogenase (LDH) release (Fig. 2D) and the number of pyknotic nuclei in the total cell population (Fig. 2E). In scrRNA-treated neurons, the reduction in mitochondrial membrane potential caused by 1 h OGD was not followed by a significant decrease in neuronal viability even after 24 h (Fig. 2D). In contrast, LDH release in Mfn2 knockdown neurons was significantly increased 24 h after OGD and exceeded 50% of the maximum possible LDH release. (Fig. 2D). The percentage of pyknotic nuclei in Mfn2 knockdown neurons 24 h after the OGD reached over 50% which was also significantly higher than in wt and scrRNA-transduced neurons (Fig. 2E).

Admittedly, the Mfn2 knockdown alone did not affect the viability of neurons during the culturing but it increased neuronal sensitivity to oxygen and glucose deprivation observed 24 h after the insult.

### Mfn2 is Necessary for the proper Mitochondrial Network Morphology and Recovery after OGD and Reoxygenation

Considering the role of Mfn2 in mitochondrial fusion and trafficking, we next analyzed the differences in the morphology of the mitochondrial network in Mfn2 knockdown neurons in response to OGD/R insult.

**Fig. 3:**
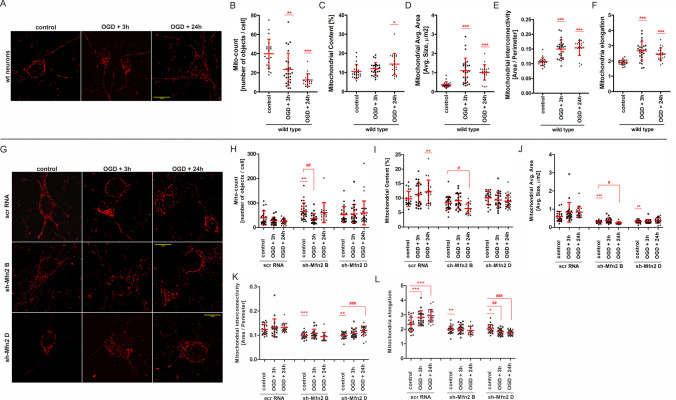
Mfn2 knockdown affects the mitochondrial network in control neurons and prevents the remodelling of mitochondrial network after OGD. (A) Representative images of mitochondria in wild-type (wt) neurons, visualized with Mitotracker Red probe, before and after OGD/R. Scale bars represent 10 μm. (B–F) Quantitative image analysis of mitochondrial network parameters in wt neurons, *n* = 3. A number of cells analyzed for each experimental point: 21–27. * *p* < 0.05, **p < 0.01, ***p < 0.001 vs wt control. (G) Representative images of mitochondria in shRNA-transduced neurons, visualized with Mitotracker Red probe, before and after OGD/R. Scale bars represent 10 μm. (H–L) Quantitative image analysis of mitochondrial network parameters in shRNA-transduced neurons, *n* = 3. A number of cells analyzed for each experimental point: 21–27. * *p* < 0.05, **p < 0.01, ***p < 0.001 vs scrRNA control; #*p* < 0.05, ##*p* < 0.01, ###*p* < 0.001 vs sh-Mfn2 control. For the image analysis, the following parameters were used: mito-count (B, H), mitochondrial content (C, I), average mitochondrial size (D, J), mitochondrial interconnectivity (E, K) and mitochondrial elongation (F, L)

Mitochondria were labelled with Mitotracker Red probe and mitochondrial morphology analysis in the soma of rat cortical neurons was performed using 2D confocal images (Fig. 3A, G). To characterize the mitochondrial network morphology, the following parameters were used: mito-count (Fig. 3B, H), mitochondrial content (Fig. 3C, I), average mitochondrial size (Fig. 3D, J), mitochondrial interconnectivity (Fig. 3E, K) and mitochondrial elongation (Fig. 3F, L) according to Dagda et al. (2009) [36].

A rapid drop in the mito-count parameter was observed in wt neurons in response to OGD/R (Fig. 3B). It was accompanied by a significant increase in mitochondrial content (Fig. 3C) and mitochondrial average size (Fig. 3D). A mito-count parameter represents the number of closely connected mitochondria recognized by the software as separate objects. A mito-count decrease, together with an elevated average mitochondria size indicates an intensified mitochondrial fusion induced by OGD/R. This is further supported by a significant increase in mitochondrial interconnectivity (Fig. 3E) and mitochondrial elongation parameters (Fig. 3F), which is initiated soon after the insult. As the reduced number of mito-objects was accompanied by the increased mitochondrial content, the flux of mitochondria to the soma in response to OGD/R may be considered.

Similar alterations in mitochondrial morphology were observed for scrRNA-transduced neurons after OGD/R (Fig. 3G). The number of mito-objects represented by the mito-count parameter was slightly reduced by the OGD/R (Fig. 3H). It was accompanied by a higher content of mitochondria (Fig. 3I) and an increased mitochondrial elongation parameter at 3 h and 24 h after the insult (Fig. 3L). However, the mitochondrial interconnectivity parameter in scrRNA control was only slightly elevated when compared to wt neurons. Such an increase was sustained after OGD/R (Fig. 3K).

By contrast, in sh-Mfn2 controls, the mito-count parameter demonstrated a significantly greater number of mito-objects than in scrRNA neurons (Fig. 3H), while the average size of mitochondria in sh-Mfn2 controls was significantly diminished (Fig. 3J). It was accompanied by a decreased mitochondrial elongation parameter (Fig. 3L) and mitochondrial interconnectivity (Fig. 3K). Therefore, the mitochondrial network in Mfn2 knockdown neurons was much more dispersed and fragmented into smaller mitochondria in comparison to scrRNA neurons, even before the insult.

Furthermore, neither the average mitochondrial size (Fig. 3J) nor the mitochondrial content (Fig. 3I) was observed to increase in Mfn2 knockdown neurons in response to OGD/R. The mitochondrial elongation parameter was lowered even more by OGD/R (Fig. 3L). Thus, the Mfn2 knockdown prevented the OGD/R-induced mitochondria gathering in the soma and mitochondria elongation observed for wt and scrRNA-transduced neurons.

As shown by the mito-count parameter, the number of identified separate mito-objects in sh-Mfn2 B neurons decreased 3 h after OGD/R, but returned to the control value 24 h later (Fig. 3H). This was followed by a transient and slight increase in the average mitochondrial size in sh-Mfn2 B neurons, as a significant decrease in this parameter was observed 24 h after OGD/R (Fig. 3J). These observations might indicate an ineffective attempt at the mitochondrial fusion in response to OGD.

All in all, wt neurons demonstrated an increased mitochondrial fusion and an enhanced mitochondrial network branching in response to OGD. The changes in the mitochondrial network started shortly after the insult and lasted up to 24 h. In sh-Mfn2 neurons, in turn, the Mfn2 knockdown altered the mitochondrial network morphology, favouring its fragmentation and preventing the post-insult remodelling of the mitochondrial network.

### Mfn2 Knockdown Enhances Mitophagy in Primary Rat Cortical Neurons

Having regard to recent reports on the relationship between mitochondrial network dynamics and mitophagy, we further examined the effect of Mfn2 knockdown on the onset of mitophagy after OGD/R.

**Fig. 4:**
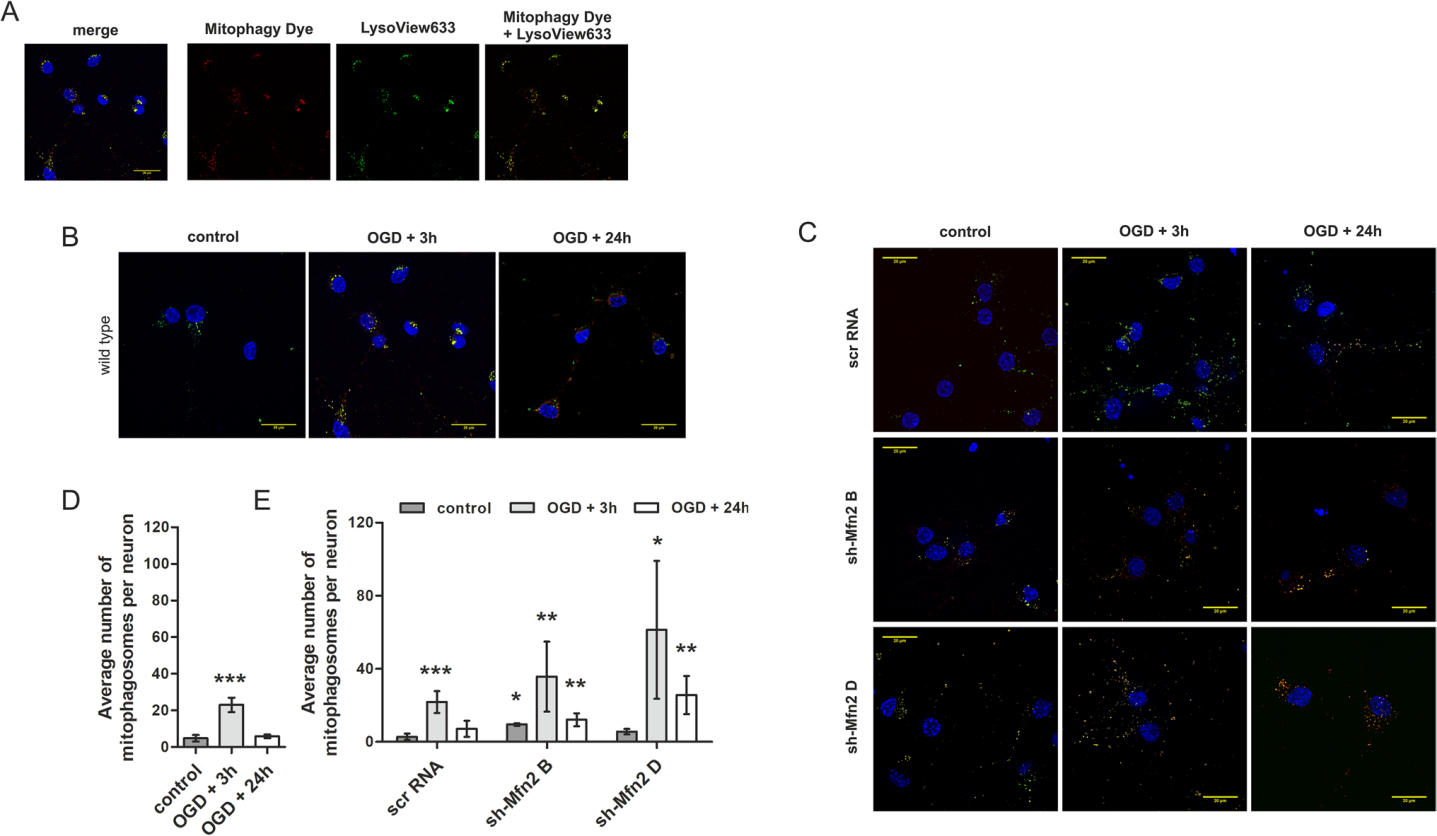
Mfn2 knockdown in primary neurons enhances late mitophagosomes formation in response to OGD/R. (A) Representative images of mitophagosomes visualized with Mitophagy Dye (red) and LysoView 633 (green) in wild-type (wt) neurons subjected to OGD/R. Representative images present: mitochondria/mitophagosomes (red), lysosomes (green) and nuclei (blue) followed by merged image. Bar: 20 μm. (B, C) Representative merged images of mitophagosomes in wt and shRNA-transduced neurons subjected to OGD/R, respectively. Mitophagosomes are seen as bright orange dots. Bar: 20 μm. (D, E) Average number of mitophagosomes per neuron in wt and shRNA neurons respectively; the number of cells in each time point = 13–32; *n* = 3–5; *p < 0.05, **p < 0.01, ***p < 0.001 vs control or scrRNA control

We used the mitochondrial-specific fluorescent probe, Mitophagy Dye, which is characterized by the shift in fluorescence intensity in response to the change in pH caused by the fusion of mitochondria-containing autophagosomes and lysosomes (Fig. 4A). To support the recognition of late mitophagosomes, lysosomes were also visualized. In the merged images, the mitochondria-lysosomes colocalization points were observed as bright orange dots in wt neurons (Fig. 4B) and shRNA-transduced neurons (Fig. 4C).

The quantitative analysis of mitophagosomes showed an increased number of late mitophagosomes in all types of neurons, shortly after OGD/R (Fig. 4D, E). However, in wt and scrRNA-transduced neurons, the number of mitophagosomes returned to control values within 24 h, indicating a transient activation of mitochondrial degradation. In Mfn2 knockdown neurons, the number of late mitophagosomes was still elevated 24 h after OGD/R (Fig. 4E).

**Fig. 5.**
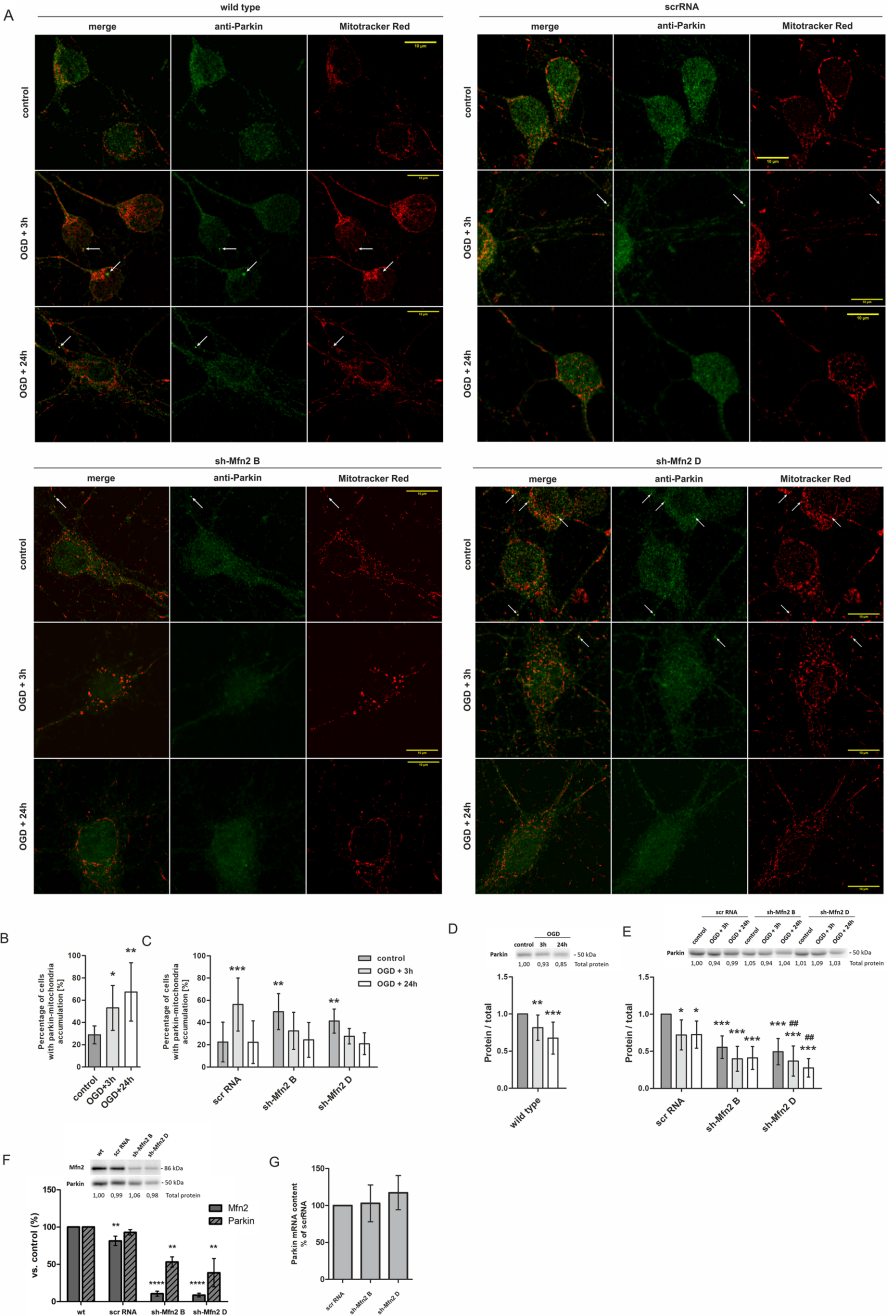
Mfn2 knockdown in neurons is accompanied by increased Parkin accumulation on mitochondria. A. Representative images of wild-type (wt), scrRNA and sh-Mfn2 neurons presenting mitochondria and Parkin accumulation (bright dots indicated by arrows). Mitochondria visualized in red (Mitotracker Red) and Parkin (anti-Parkin) pseudocoloured in green. Bar = 10 μm. (B, C) Average percentage of wt and shRNA-transduced neurons positive for Parkin and mitochondria accumulation in controls and after OGD/R, *n* = 3. *p < 0.05, **p < 0.01, ***p < 0.001 vs wt or scrRNA controls. (D, E) Representative western blots and densitometric analysis of Parkin in cell lysates of wt and shRNA-transduced neurons, respectively, after OGD/R, *n* = 5. (F) Representative western blot and densitometric analysis of Parkin level in cell lysates of control wt, scrRNA- and sh-Mfn2 neurons on DIV10; *n* = 4–6. (G) Quantitative Real-Time PCR for Parkin in scrRNA- and sh-Mfn2 neurons on DIV10; *n* = 3, *n* = 3. The optical density of the particular bands on western blots was normalized to total protein in line stained with Ponceau S and is presented relative to wt or scrRNA controls as mean ± SD; Normalization factors are shown under representative western blot images. *p < 0.05; **p < 0.01, ****p < 0.0001 vs wt (B, D, F) or scrRNA control (C, E). #*p* < 0.05, ##*p* < 0.01, vs sh-Mfn2 control (E)

The study on mitophagosomes formation was followed by an analysis of E3-ubiquitin ligase Parkin accumulation on mitochondria. An increased recruitment of Parkin to mitochondria is considered as one of the hallmarks of mitochondria damage and a crucial step in selective mitochondria elimination. Immunocytochemical staining was performed for Parkin along with mitochondria labelling with Mitotracker Red probe. Confocal images were taken for wt and shRNA-transduced neurons (Fig. 5A).

The quantitative analysis of merged confocal images revealed an increase in the percentage of neurons with Parkin accumulation on mitochondria in wt and scrRNA neurons 3 h after the OGD/R (Fig. 5B, C). It was accompanied by a drop in Parkin protein levels in total cell lysates of wt (Fig. 5D) and scrRNA neurons (Fig. 5E). However, in Mfn2 knockdown neurons, Parkin accumulation on mitochondria was significantly elevated even before OGD (Fig. 5C) and it was accompanied by a decreased Parkin protein level in sh-Mfn2 controls, reaching only 53% and 39% (for shMfn2 B and shMfn2 D respectively) of wt controls (Fig. 5E, F). Further analysis by quantitative RT-PCR confirmed an equal expression of Parkin mRNA in scrRNA and sh-Mfn2 neurons, which excluded the presumption that the differences in Parkin protein levels between the controls might result from shRNA off-target effects (Fig. 5G).

Summing up, an increased Parkin localisation on mitochondria along with decreased Parkin protein levels in sh-Mfn2 control neurons suggest a mitochondrial dysfunction induced by Mfn2 knockdown (Fig. 5). Moreover, the Mfn2 knockdown resulted in a prolonged mitochondrial elimination in response to OGD/R, as evidenced by the increased number of mitophagosomes in sh-Mfn2 neurons 24 h after the insult (Fig. 4).

### Mfn2 Knockdown Impairs Compensatory Mitochondrial Biogenesis in Rat Cortical Neurons Subjected to OGD/R

Since mitochondria are the main source of energy in neurons, further investigation was aimed at establishing whether mitochondrial damage caused by OGD/R could be alleviated by compensatory mitochondrial biogenesis also in Mfn2 knockdown neurons.

**Fig. 6:**
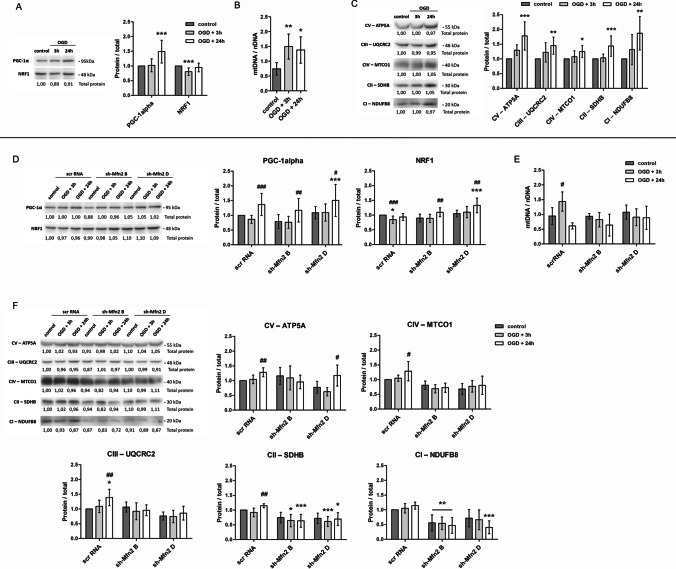
Mfn2 knockdown supresses biosynthesis of respiratory chain proteins in neurons after OGD/R. (A, D) Representative western blots and densitometric analysis of mitochondria biogenesis key proteins: PGC-1α and NRF-1 in cell lysates of wild-type (wt) and shRNA-transduced neurons, respectively, *n* = 5. (B, E) Average mitochondrial to nuclear DNA ratio (mtDNA/nDNA) in wt and shRNA neurons, respectively, before and after OGD/R; Data are expressed as mean ± SD, *n* = 5. (C, F) Representative western blots and densitometric analysis of representative respiratory chain subunits in cell lysates of wt and shRNA neurons, respectively, *n* = 5. Western blot data are shown as the fold change over control of the protein of interest. Optical density of particular bands was normalized to total protein in line stained with Ponceau S and presented as mean ± SD. The normalization factors are shown under representative western blot images. * *p* < 0.05, **p < 0.01, ***p < 0.001 vs wt control or scrRNA control; #*p* < 0.05, ##*p* < 0.01, ###*p* < 0.001 vs sh-Mfn2 control

As shown by western blot analysis, the level of PGC-1α, the upstream regulator of mitochondrial biogenesis, was significantly increased in all the considered types of neurons 24 h after the insult (Fig. 6A, D). The downstream factor NRF-1 was significantly increased in sh-Mfn2 neurons (Fig. 6D), while in wt and scrRNA neurons, the level of NRF-1 was transiently decreased (Fig. 6A, D).

Western blot analysis was supported by the measurement of mitochondrial DNA content (Fig. 6B, E). An increased mitochondrial to nuclear DNA ratio (mtDNA/nDNA) was observed in wt neurons shortly after the insult (Fig. 6B). It was followed by a significant rise in the protein level of the representative subunits of the respiratory chain complexes 24 h after the insult (Fig. 6C). A similar relationship was observed for scrRNA neurons. However, an increase in mtDNA/nDNA ratio was transient (Fig. 6E) and the following increases of particular respiratory chain subunits were also present, yet not as pronounced as for wt neurons (Fig. 6F).

In contrast to control neurons, no increase in the mtDNA/nDNA ratio after the insult (Fig. 6E) and no rise in protein levels in any of the respiratory chain subunits considered (Fig. 6F) were observed in Mfn2 knockdown neurons.

Thus, Mfn2 knockdown appeared to supress the respiratory chain protein biogenesis in neurons after OGD/R.

## Discussion

In this paper, we hypothesized that Mfn2 may act as a regulatory factor which integrates mitochondrial network dynamics with mitophagy and mitochondrial biogenesis in neurons following a transient ischemic insult.

We showed that in response to OGD/R, the dynamics of the mitochondrial network in wt neurons was increased to ameliorate the mitochondrial damage caused by the lack of oxygen and glucose. In addition, to prevent the bioenergy failure, compensatory biosynthesis of the respiratory chain proteins was initiated. However, in Mfn2 knockdown neurons, mitochondrial damage was not repaired by mitochondrial network dynamics. We demonstrated that Mfn2 knockdown prevented the remodelling of the mitochondrial network favouring the fragmentation of the mitochondria, intensified mitochondrial elimination and prevented the biosynthesis of respiratory complexes following OGD/R. Consequently, an increased neuronal death after OGD/R was observed for Mfn2 knockdown neurons, strongly suggesting that Mfn2 is one of the essential elements of the neuronal response to ischemic insult, necessary for the neuronal survival.

Alterations in mitochondrial dynamics have been implicated in many neurodegenerative diseases [1, 44]. Previous studies showed that Mfn2 was involved in mitochondrial trafficking in axons [45] and the abolition of this function of Mfn2 by genetic mutations or knockout changed mitochondrial movement and lead to axonal degeneration as a consequence [46]. Moreover, a considerable number of experimental models have observed a link between mitochondrial fusion and fission and the onset of mitophagy [47, 48]. Multiple studies demonstrated Drp1-driven mitochondrial fission as a frequent consequence of ischemic injury [6]. According to Zuo et al. (2014), Drp1-driven mitochondrial fission contributed to neuronal survival by supporting the elimination of damaged mitochondria [47], while Kumar et al. (2016) indicated the biphasic mitochondrial fragmentation profile. In their study, OGD was followed by an extensive mitochondrial fragmentation which preceded apoptosis and neuronal death or by a moderate mitochondrial fragmentation followed by an increase in mitochondrial fusion during the re-oxygenation phase, resulting in neuronal survival [49]. In primary neurons, Nair et al. (2022) found a primary mitochondria fission wave immediately after 90-min OGD with a significant increase in mitophagy followed by a secondary phase of fission at 24 h following recovery [50]. Although it was hypothesised that excessive mitophagy in the early phase was a pathologic response which may contribute to secondary energy depletion, secondary mitophagy may be involved in regeneration and repair [50]. Further studies reported that increased events of mitochondrial fusion and fission supported the maintenance of mitochondrial function and thus cell survival by enhanced mixing of mitochondrial content [51]. The post-OGD intensified mitochondrial fusion in wt neurons observed in our model is consistent with the previous findings as it also seemed to contribute to neuronal survival.

In our study, the mitochondria in Mfn2 knockdown neurons showed a decreased size followed by reduced mitochondrial interconnectivity and elongation. As mentioned earlier, mitochondrial fusion and trafficking may also be mediated by Mfn2 homologue, Mfn1, which has been recorded to show even greater fusion activity [11]. However, in our model, selective Mfn2 knockdown was sufficient to alter mitochondrial morphology before the insult and to suppress mitochondrial network remodelling in response to OGD/R. Meanwhile, the protein levels of other agents mediating mitochondrial dynamics, Opa1 and Drp1, were not changed by shRNA transduction (data not shown) and Mfn1 was elevated (Fig. 2B). This was followed by prolonged mitophagy and increased neuronal death, as demonstrated by the increased LDH release and a higher number of pyknotic nuclei. Our data show that Mfn2 is required in the reoxygenation phase for the proper mitochondrial recovery. We suggest that Mfn2 is necessary for mitochondrial network post-insult remodelling which serves as an early quality control mechanism, and thus prevents over-elimination of mitochondria. These observations are consistent with the report by Puri et al. (2019), who demonstrated that mitochondrial elimination in neurons was secondary towards mitochondria repair [21].

In our model, Mfn2 protein level in wt and scrRNA neurons was significantly reduced after the OGD/R. As previously shown by Wappler et al. (2013), the duration of OGD, thus the severity of the insult, resulted in different involvement of key proteins mediating mitochondrial fusion and fission. In their model, 1 h OGD did not alter the protein level of pro-fusion (Mfn1/2 and Opa1) and pro-fission (Drp1, Fis1) proteins although some alteration in mitochondrial shape and mitochondrial network were observed [24]. However, in our experimental conditions, 1 h OGD proved to be sufficient to cause mitochondrial network remodelling that was accompanied by a significant reduction of Mfn2 protein. The discrepancy may potentially result from minor differences in the experimental and culturing conditions, e.g. a different ratio between neurons and glia causing different susceptibility towards the insults.

The observed constant reduction of Mfn2 after OGD/R in wt neurons appeared to be specific for this protein, as we did not observe similar changes for its homolog, Mfn1, and the mitochondrial matrix marker, Hsp60. We did, however, observe a transient drop in Opa1, Drp1 and TOM22 immunoreactivity at 3 h. This may imply the participation of enhanced mitophagy or other cellular processes, such as e.g. proteasomal degradation of OMM proteins which may not necessarily lead to the elimination of whole mitochondria but might be involved in endogenous mechanisms regulating mitochondria elimination [52].

OGD-induced reduction of Mfn2, as was observed here, is in line with the outcomes obtained in previous studies in in vitro and in vivo models [53]. As demonstrated by McLelland et al. (2018), Mfn2 proteasomal degradation facilitated the dissociation of mitochondria from ER thereby enabling mitophagy [20]. In our model, the number of mitophagosomes in Mfn2 knockdown neurons outranked their number in wt and scrRNA neurons after OGD/R (3 h and 24 h), indicating intensified mitophagy in comparison to wt neurons. However, the increased mitochondrial elimination in Mfn2 knockdown neurons did not support cells survival. Therefore, our conclusion is that increased mitophagy alone does not contribute to neuroprotection and that Mfn2 knockdown may disrupt the balance between mitochondrial recovery and mitochondrial elimination, increasing the neuronal susceptibility towards ischemic insult.

According to recent studies, Mfn2 degradation after ischemic insult is mediated by E3 ubiquitin ligase, Parkin [18, 54]. As shown by Chen et al. (2013), PINK1 phosphorylates Mfn2 to facilitate the accumulation of Parkin on damaged mitochondria [17]. Simultaneously, Parkin mediates Mfn2 ubiquitination and degradation, which has an impact on mitochondria-ER tethering [18, 20]. Considering the above, the mutual relation between Mfn2 and Parkin seems to link mitochondrial dynamics with mitochondrial elimination. Here, we have shown that Mfn2 knockdown was accompanied by a decrease of Parkin and the reduction did not result from altered mRNA expression. It seems to support the thesis on Mfn2-Parkin relationship in mitophagy coordination. Enhanced Parkin localisation on mitochondria in sh-Mfn2 neurons together with a decreased mitochondrial membrane potential, as observed for sh-Mfn2 D neurons, may additionally indicate that Mfn2 knockdown causes mild mitochondrial dysfunction, which does not affect neuronal survival in control conditions but contributes to increased susceptibility towards OGD. The exact mechanism of this phenomenon, however, requires further investigation.

In our model, OGD/R was observed to induce an increase in PGC-1α protein level. In wt neurons, it was followed by an increase in mtDNA content and the biosynthesis of representative proteins for respiratory complexes, revealing a mitochondrial biogenesis as an integral element of pro-surviving response to OGD/R. This phenomenon was not observed in Mfn2 knockdown neurons. The mechanism is not fully understood, but several considerations have already emerged. The mtDNA particles in cells are packed into mtDNA-protein complexes, nucleoids [55], and can be passively transferred between mitochondria during mitochondrial fusion and fission events [56]. Considering the above, Mfn2 deficiency may affect mtDNA synthesis and segregation by impairing mitochondrial fusion and trafficking. It has also been demonstrated that for proper mtDNA distribution, especially in peripheral zones of the cell, nucleoids have to be actively transported via Kinesin Family Member (KIF5B)-driven mitochondrial dynamic tubulation activities (Miro) that occur predominantly at the ER-mitochondria contact sites [57]. ER-mitochondria contacts are the main location of distribution of newly synthetized mtDNA to daughter mitochondria. It can therefore be assumed that the proper mitochondria-ER positioning, as also determined by Mfn2, may be crucial for mtDNA synthesis in response to ischemic insult. More research is needed to elucidate the molecular mechanism describing how Mfn2 knockdown affects not only the mtDNA biosynthesis and distribution but also mitochondrial biogenesis in general.

## Conclusion

Our data support the hypothesis that Mfn2 in neurons is involved in their response to mild and transient OGD/R stress by balancing the rate of elimination of defective mitochondria. In addition, Mfn2 has a positive influence on the mitochondrial restoration expressed as mtDNA and proteins in the respiratory chain content. In Mfn2 knockdown neurons, stress recovery is not as efficient as in wt cells. This may potentially be caused by mitochondrial impairment which is not effectively repaired by restoration of mitochondrial network dynamics and respiratory chain protein content while mitochondria elimination is enhanced after the insult. Consequently, Mfn2 knockdown results in increased neuronal death following OGD/R stress, confirming that Mfn2 is one of essential elements of neuronal response to ischemic insult, crucial for neuronal survival.

## Supplementary Information

ESM 1 (PNG 6364 kb)

High resolution image (TIF 508695 kb)

## Data Availability

The datasets generated during and/or analysed during the current study are available from the corresponding author on reasonable request.

## Declarations

All procedures involving animals were performed in accordance with the ethical standards of the institution or practice at which the studies were conducted.
