# Supplementary figures and images for: Mitofusin 2 Integrates Mitochondrial Network Remodelling, Mitophagy and Renewal of Respiratory Chain Proteins in Neurons after Oxygen and Glucose Deprivation
Source: Mol Neurobiol. 2022 Aug 13;59(10):6502–18. doi: 10.1007/s12035-022-02981-6 (PMC9463309; doi:10.1007/s12035-022-02981-6)

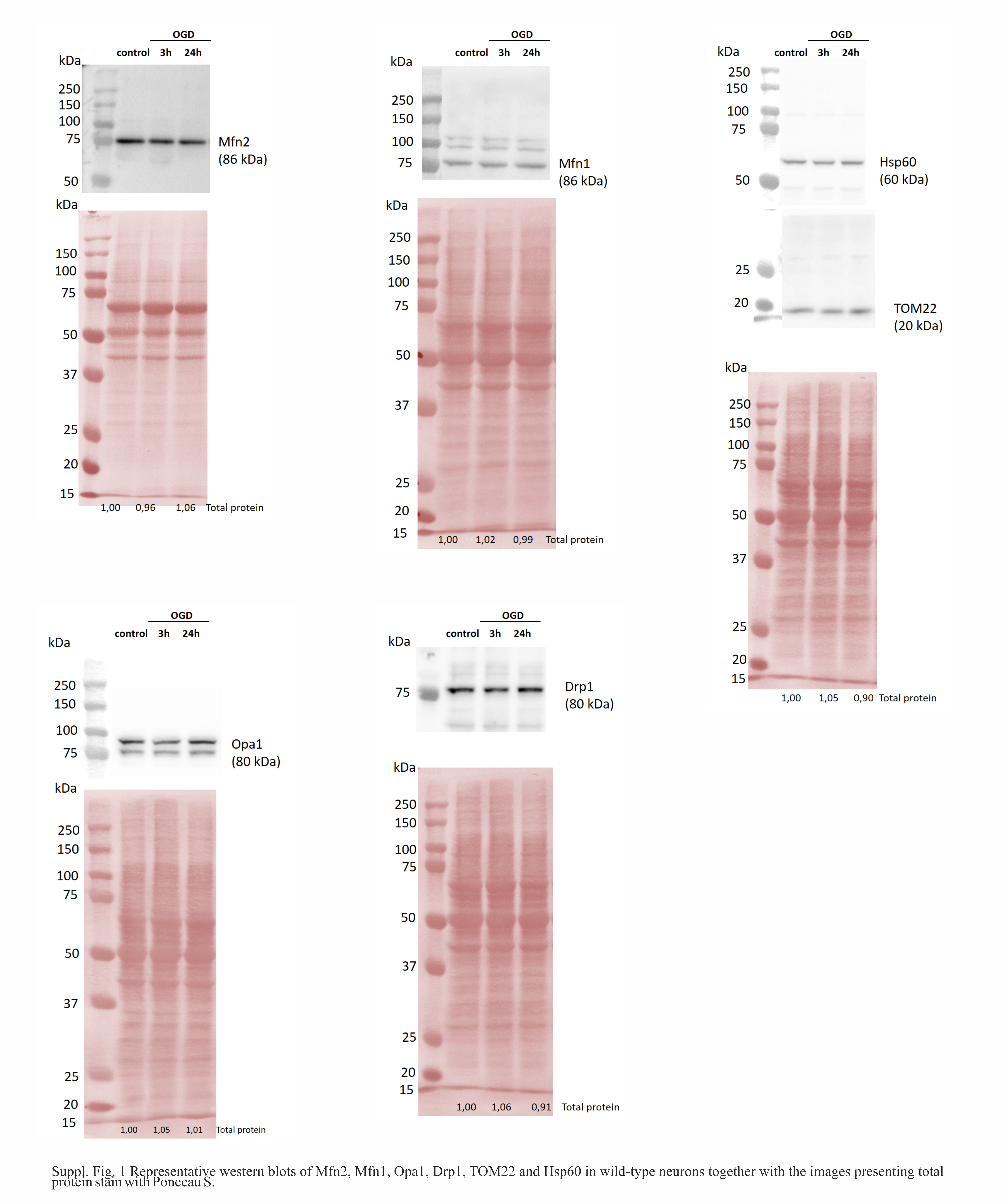

Supplement: Supplementary file 1 — (PNG 6364 kb) [file 12035_2022_2981_Fig7_ESM.png]
